# Obesity-Susceptibility Loci and Their Influence on Adiposity-Related Traits in Transition from Adolescence to Adulthood - The HUNT Study

**DOI:** 10.1371/journal.pone.0046912

**Published:** 2012-10-19

**Authors:** Koenraad Frans Cuypers, Ruth J. F. Loos, Kirsti Kvaløy, Bettina Kulle, Pål Romundstad, Turid Lingaas Holmen

**Affiliations:** 1 HUNT Research Center, Levanger, Department of Public Health and General Practice, Faculty of Medicine, Norwegian University of Science and Technology, Trondheim, Norway; 2 MRC Epidemiology Unit, Institute of Metabolic Science, Addenbrooke’s Hospital, Cambridge, United Kingdom; 3 The Charles Bronfman Institute for Personalized Medicine, Child Health Institute, Genetics of Obesity and Related Metabolic Traits Program, Mount Sinai School of Medicine, New York, New York, United States of America; 4 Epi-Gen, Institute of Clinical Medicine, Akershus University Hospital, University of Oslo, Oslo, Norway; 5 Department of Biostatistics, Institute of Basic Medical Sciences, University of Oslo, Oslo, Norway; 6 Department of Public Health and General Practice, Faculty of Medicine, Norwegian University of Science and Technology, Trondheim, Norway; Sanjay Gandhi Medical Institute, India

## Abstract

**Introduction:**

Obesity-susceptibility loci have been related to adiposity traits in adults and may affect body fat estimates in adolescence. There are indications that different sets of obesity-susceptibility loci influence level of and change in obesity-related traits from adolescence to adulthood.

**Objectives:**

To investigate whether previously reported obesity-susceptible loci in adults influence adiposity traits in adolescence and change in BMI and waist circumference (WC) from adolescence into young adulthood. We also examined whether physical activity modifies the effects of these genetic loci on adiposity-related traits.

**Methods:**

Nine obesity-susceptibility variants were genotyped in 1 643 adolescents (13–19 years old) from the HUNT study, Norway, who were followed-up into young adulthood. Lifestyle was assessed using questionnaires and anthropometric measurements were taken. The effects of genetic variants individually and combined in a genetic predisposition score (GPS) on obesity-related traits were studied cross-sectionally and longitudinally. A modifying effect of physical activity was tested.

**Results:**

The GPS was significantly associated to BMI (B: 0.046 SD/allele [0.020, 0.073], p = 0.001) in adolescence and in young adulthood (B: 0.041 SD/allele [0.015, 0.067], p = 0.002) as it was to waist circumference (WC). The GPS was not associated to change in BMI (p = 0.762) or WC (p = 0.726). We found no significant interaction effect between the GPS and physical activity.

**Conclusions:**

Our observations suggest that obesity-susceptibility loci established in adults affect BMI and WC already in adolescence. However, an association with change in adiposity-related traits from adolescence to adulthood could not be verified for these loci. Neither could an attenuating effect of physical activity on the association between the obesity-susceptibility genes and body fat estimates be revealed.

## Introduction

During the last decades the prevalence of overweight and obesity have increased worldwide in adolescents and adults, which has been largely attributed to lifestyle changes [1]–[3]. However, twin and family studies have estimated that also genetic factors contribute to the inter-individual variation in the susceptibility to obesity. Heritability estimates range from 40 to 70% [4], with suggestive evidence for higher estimates earlier in life [5]–[7]. BMI in adolescence tends to track into adulthood, hence obese and overweight adolescents are likely to be obese or overweight adults [8], [9]. While some have shown absence of genetic contribution to change in BMI [10], others have suggested that BMI level and change are influenced by different genes [11], [12].

So far, large-scale meta-analyses of GWAS have identified 32 loci robustly associated with BMI [13]–[19] in adults, and evidence that these loci affect BMI in childhood and adolescence is growing [5], [6], [17], [20]. A recent meta-analysis including 13 071 children and adolescents found that the combined effect of the 12 BMI loci studied was similar to the effect seen in adults [21]. However, when loci were considered individually, effect sizes tended to differ, with sometimes larger effects (variants near *SEC16B*, *TMEM18*, and *KCTD15*) and sometimes smaller effects (variant near *BDNF*) in children and adolescents than in adults [21], suggesting that some loci might affect weight change throughout the life course. The evidence that these loci are indeed associated with change in BMI from childhood and adolescence into adulthood is limited. Some suggest that the effects of *FTO* and *MC4R* loci increase during adolescence reaching a maximum at age 20 years, followed by a slow but continuous decrease into late adulthood [5]. Others have observed association between the *FTO* and BMI in childhood, but only after an inverse association between this variant and BMI in infancy [7]. So far, the effect of other loci on change in BMI from adolescence to adulthood has not yet been examined.

We genotyped nine loci (near or in *FTO, MC4R, TMEM18, GNPDA2, NEGR1, BDNF, KCTD15, MTCH2, and TFAP2B*), previously shown to be significantly associated with adult BMI [13], to study the effect on the level of BMI and waist circumference (WC) cross-sectionally in adolescence respectively adulthood and to investigate the effect on changes in BMI and WC from adolescence into young adulthood. Our study included data from 1 643 adolescents (13–19 years) from the HUNT study (Norway) who were followed-up into young adulthood 11 years later. Loci were considered individually as well as combined in a genetic predisposition score (GPS). Furthermore, we examined whether the association between the loci and obesity-susceptibility was modified by physical activity.

## Methods

### Study Population

During the last 25 years the Health Study of Nord-Trøndelag, HUNT, a large population- based study (http://www.ntnu.no/hunt), has been carried out three times in the county of Nord-Trøndelag (130 000 inhabitants), Norway, a homogenous population of white-European descent. The population’s geography, demography and occupational activities are fairly representative for the whole of Norway, though it lacks large cities [22]. Although the average income and educational levels are slightly lower than the national average, the socioeconomic inequality in mortality in the region is at the national level [23].

All students in junior and senior high schools (13–19 years) in the county were invited to participate in Young-HUNT, the adolescent part of HUNT. Young-HUNT1, which formed the basis of the current study, was carried out between 1995 and 1997 and recruited a total of 8 408 adolescents (response rate 83%) who participated by undergoing a health examination and by completing the questionnaires. Our final study-sample included the 1 643 participants who participated both as adolescents in Young-HUNT1 and as young adults (23–29 years) 11 years later in HUNT 3, had complete sets of phenotypic data, good quality genotype data and who were not pregnant at follow-up (Table S1–S2).

At both time points, all participants completed comprehensive self-administered questionnaires on physical and mental health, somatic complaints and lifestyle and were clinically examined by trained nurses using the same protocols for anthropometric measures. Blood samples were drawn at young adulthood in HUNT3.

### Measurements

Weight and waist circumference were obtained by using standardized weight scales and meter bands. Height was measured by trained nurses using internally standardized measuring tapes. The participants wore light clothing (as T-shirts and trousers) and were barefoot. Height was measured to the nearest centimetre (cm) and weight to the nearest 0.5 kilogram (kg). BMI, as a measure of overall adiposity, was calculated as weight (kg) divided by squared height (m^2^). Waist circumference, a measure for central adiposity, was measured to the nearest centimetre applying non-stretchable band horizontally at the umbilical level after the participants emptied their lungs, or midway between the last rib and the iliac cristae if the latter was larger.

Overweight (n = 260) and obesity (n = 35) in adolescents was defined by standardising BMI in relation to sex and age using the reference growth charts as proposed by the International Obesity Task Force (IOTF) [24]. In adults, the WHO cut-offs were used; i.e. normal weight as 18.5 kg/m^2^<BMI<25 kg/m^2^, overweight as BMI≥25 kg/m^2^ (n = 775) and obese as BMI≥30 kg/m^2^ (n = 265).

Because of the age differences among adolescents and in order to compare data in adolescence and adulthood, we standardized BMI and waist circumference distributions to a mean of 0 and a SD of 1 by calculating age-and-sex-specific z-scores at baseline and sex-specific z-scores at follow-up. BMI change in z-scores was calculated as the difference between the z-scores in adulthood and in adolescence.

Pubertal status was assessed using the Pubertal Development Scale (PDS) [25], which is based on self-reported ratings of participants’ growth spurt and pubic hair growth. In addition, status of menarche and breast development was assessed in girls and breaking of the voice and growth of facial hair in boys. Each item was scored from 1 (have not begun) to 4 (completed), except for menarche, which was reported as a dichotomous variable (coded 1: have not begun and 4: completed). Pubertal status was calculated as the average of all scores.

Physical activity was assessed by questionnaires as described previously for adolescents [26] and adults [27].

### SNP Selection

At the time of the design of the study (2009), nine of the currently more than 50 established obesity-susceptibility loci (near *BDNF,* near *MC4R,* near *TFAP2B*, in *FTO*, near *NEGR1*, near *TMEM18*, in *MTCH2*, near *GNPDA2,* near *KCTD15*) had been reported to be robustly associated with BMI. We subsequently decided to not genotype the more recently identified loci as these had been identified with large-scale meta-analyses, such that our study would be most definitely insufficiently powered to confirm associations in our population.

Nine SNPs representing nine obesity-susceptibility loci identified by GWA studies [14]–[19], [28] were selected for analyses; i.e. near *BDNF* (rs4074134), near *MC4R* (rs17782313), near *TFAP2B* (rs987237), in *FTO* (rs1121980), near *NEGR1* (rs2815752), near *TMEM18* (rs6548238), in *MTCH2* (rs10838738), near *GNPDA2* (rs10938397), and near *KCTD15* (rs11084753).

### Genotyping

All markers were genotyped using a Sequenom iPlex assay on a MassARRAY platform (Sequenom, San Diego, CA) at CIGENE, Centre for Integrative Genetics, The Norwegian University of Life Sciences in ÅS, Norway. All variants passed initial quality-control criteria with a call-rate ≥95% and genotype distribution in Hardy-Weinberg equilibrium (P>0.05) (Table 1).

**Table 1:** Genotype information and quality control statistics for the 9 obesity-susceptibility variants included in the study.

SNP	Chrom.	Position	Nearest gene	Effect allele	Effect allele	Other allele	References	Call rate	Genotype freq			HWE	
					frequency				Risk allele homozygous	Heterozygous	Other allele homo	p-value	
					%				%	%	%		
rs2815752	1	72585028	NEGR1	A	59.2	G	1,6	99.7	34.5	49.3	16.2	0.525	
rs6548238	2	624905	TMEM18	C	83.7	T	1,6	99.4	69.9	27.5	2.6	0.669	
rs10938397	4	44877284	GNPDA2	G	38.8	A	1,6	99.4	15.2	47.3	37.6	0.765	
rs987237	6	50911009	TFAP2B	G	17.7	A	9	98.4	3.3	28.8	67.9	0.745	
rs10838738	11	47619625	MTCH2	G	36.3	A	1,6	99.6	13.9	44.7	41.4	0.221	
rs4074134	11	27603861	BDNF	G	81.5	A	7,8	99.7	66.4	30.2	3.4	0.752	
rs1121980	16	52366748	In FTO	A	44.4	G	1,3	99.7	18.9	51.0	30.1	0.139	
rs17782313	18	56002077	MC4R	C	26.9	T	4	98.4	6.7	40.4	52.9	0.203	
rs11084753	19	39013977	KCTD15	G	69.5	A	1,6	99.2	47.6	43.7	8.7	0.181	

Article reference: 1) Loos et al., 2009; 2) Hinney et al., 2007; 3) Loos et al., 2008; 4) Willer et al., 2009; 5) Zhao et al., 2009; 6) Thorleifson et al., 2009;

HWE: Hardy-Weinberg equilibrium; Call-rate: rate of successful genotyping. All variants passed initial quality-control criteria with a call-rate ≥95% and genotype distribution were in Hardy-Weinberg equilibrium (P>0.05). The genotype distribution and effect allele frequencies varied from 17.7% for rs987237 to 83.7% for rs654238), which were in consistency with previous reports.

### Statistical Analyses

Genotypes were coded 0, 1 or 2 according to the number of BMI-increasing alleles for each SNP, with the BMI-increasing alleles defined by the results reported by previous GWA studies. [13], [16], [17], [28] For estimating the effect of the SNPs combined, we calculated a genetic predisposition score (GPS) by summing the BMI-increasing alleles across the nine SNPs. Individuals with missing genotypes for more than three SNPs (n = 9) were excluded for the calculation of the GPS. For individuals with missing genotypes for three or fewer SNPs (n = 53), missing genotypes were replaced with the average allele count of the respective SNP for the purpose of calculating the genetic predisposition score. The GPS calculated for the 1634 individuals was normally distributed (mean number of effect alleles = 9.17, SD = 1.86 and median number of effect alleles = 9.00). We did not weight the risk alleles on basis of their individual effect sizes because no well-accepted effect sizes were available for each of the SNPs, and it has been shown that weighting of risk alleles may have limited effect on the results [29].

Cross-sectional analyses: We tested for association between individual SNPs and the GPS with the z-scores of BMI and WC in adolescence and in young adulthood using linear regression analyses, assuming an additive effect of the BMI-increasing allele. Analyses with BMI and WC in adolescence were adjusted for pubertal development, while age was adjusted for in young adulthood. Analyses with WC as outcome were in addition adjusted for height in both adolescents and adults.

Following regression equations show the use of different adjustments in the various models.

Associations of the individual obesity-susceptibility SNPs and the GPS with adiposity-related traits in adolescence:
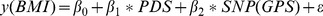


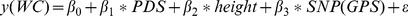



Associations of the individual obesity-susceptibility SNPs and the GPS with adiposity-related traits in adulthood:
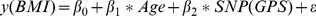


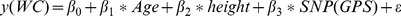



We used logistic regression to test for associations between the GPS and risk of obesity and overweight in adolescence and in adulthood. Analyses in adolescence were adjusted for sex, age, and pubertal development score and in adulthood for sex and age in the model with BMI as outcome and additionally adjusted for height in the model with waist circumference as outcome.

Longitudinal analyses: We tested the association between each SNP and the GPS with change in BMI and WC from adolescence into young adulthood, using linear regression analyses assuming an additive effect for the BMI-increasing allele. Analyses were adjusted for PDS in adolescence and for age-difference between baseline and follow-up. Change in WC was additional adjusted for adult-height. Associations of the individual obesity-susceptibility SNPs and the GPS with change in adiposity-related traits from adolescence into adulthood:
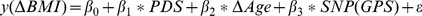


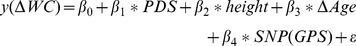



Interactions with physical activity for both the cross-sectional and longitudinal models were tested by including the cross-product term (physical activity respectively in adolescence and adulthood*SNP and physical activity respectively in adolescence and adulthood* GPS) in the model, in addition to the main effect of the SNP or GPS and physical activity.

Statistical analyses were performed using PLINK and SPSS Statistics, version 18. All tests were performed using the nominal level of significance of p = 0.05.

### Ethics

All participants and guardians of adolescents younger than 16 years signed an informed consent to participation and use of data in research. Participation in the HUNT study was voluntary and approved by the Norwegian Data Inspectorate, the Directorate of Health and recommended by the Regional Committee for Medical Research Ethics (REK-2009/740-2), who also approved the present study. The study has been conducted according to the principles expressed in the Declaration of Helsinki.

## Results

### Cross-sectional Analyses

**Figure 1:**
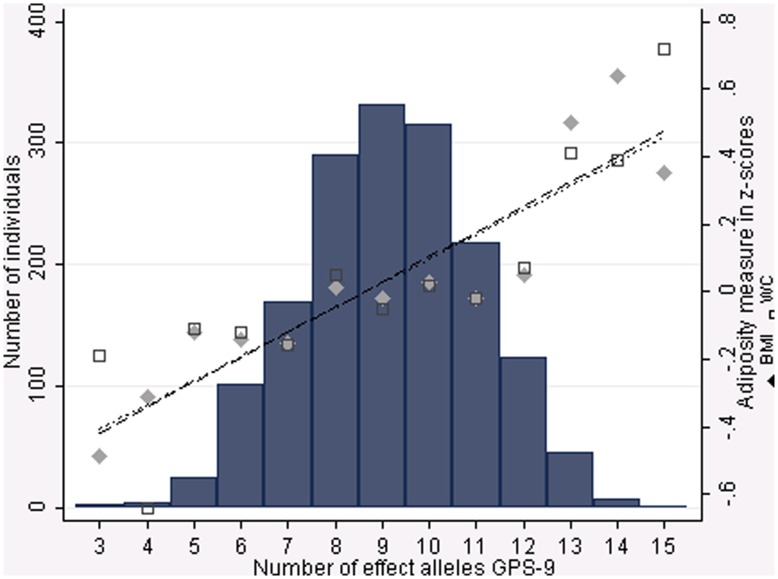
Distribution of the genetic predisposition score (GPS) and the cumulative effects of risk alleles from the nine variants on BMI z-scores and Waist circumference z-scores. The genetic predisposition score (GPS, n: 1 634 adolescents) was constructed by summing the effect alleles of each SNP ( = BMI-increasing alleles defined in the original genome-wide association studies). (rs4074134 near *BDNF*, rs17782313 near *MC4R*, rs987237 near *TFAP2B*, rs1121980 in *FTO*, rs2815752 near *NEGR1*, rs6548238 near *TMEM18*, rs10838738 in *MTCH2*, rs10938397 near *GNPDA2*, rs11084753 near *KCTD15*).

Characteristics of the adolescents and young adults (N, mean, SD) stratified by sex are shown in Table S1. In adolescence, six of the nine BMI-loci showed associations with BMI and WC directionally consistent with earlier reports [17], [20], [21]. Only associations with the loci near *MC4R*, and near *KCTD15* reached significance with BMI, while only the locus near *MC4R* showed significant association with WC. The genetic predisposition score (GPS) was significantly associated with both BMI (B: 0.046 SD/allele [CI95%: 0.020, 0.073], p = 0.001) and WC z-score (B: 0.038 SD/allele [CI95%: 0.012, 0.064], p = 0.005) (Table 2; Figure 1). Each additional BMI-increasing allele in the GPS was associated with a 1.11-fold [CI95%: 1.03, –1.19], p = 0.009 increase in the odds of overweight and obesity compared to normal-weight.

**Table 2:** Associations of the individual obesity-susceptibility SNPs and the GPS^1^ with adiposity-related traits^2^ in adolescence of Young-HUNT1^3^.

			Z-scores BMI				Z-scores WC			
SNP	Chrom	Nearest gene	B	CI (95%)	P-value	Literature	B	CI (95%)	P-value	Literature
rs2815752	1	NEGR1	−.019	−.091, .052	.596	0.01^c^	−.010	−.082, .061	.780	0.062^a^
rs6548238	2	TMEM18	.074	−.020, .168	.124	0.10^a^	.071	−.023, .165	.137	0.068^a^
rs10938397	4	GNPDA2	−.004	−.075, .067	.920	0.06^a^	.019	−.051, 090	.591	0.041^a^
rs987237	6	TFAP2B	.040	−.050, .130	.387	0.069^a^	−.004	−.094, .086	.935	0.056^a^
rs4074134	11	BDNF	.026	−.063, .114	.571	0.03^a^	.021	−.068, .110	.642	0.034^a^
rs10838738	11	MTCH2	−.021	−.092, .050	.569	0.03^c^	−.030	−.100, .041	.411	0.001^a^
rs1121980	16	inFTO	.057	−.013, .128	.110	0.06^b^	.070	.000, .140	.052	0.004^a^
rs17782313	18	MC4R	.177	.098, .256	.0001	0.07^a^	.155	.076, .235	.0001	−0.006^a^
rs11084753	19	KCTD15	.107	.030, .183	.006	0.05^a^	.053	−.023, .130	.172	0.009^a^
GPS			.046	.020, .073	.001	0.044^a^	.038	.012, .064	.005	0.025^a^

1 The genetic predisposition score (GPS) is the sum of effect alleles from each of the nine individual SNPs.

2 Age and sex specific z-scores of BMI and waist circumference in adolescence.

3 Number of participants: for individual SNPs = 1643 and for GPS = 1634 (those missing more than 3 SNPs excluded).

The linear regression models were adjusted for pubertal maturity regarding BMI and additionally also for height regarding WC, assuming an additive effect. Pregnant participants were excluded.

Chrom: chromosome.

Comparable effect sizes in the literature:

a den Hoed et al. [21];

b Zhao et al.

c Willer et al. [17].

In young adulthood, the association of seven and six of the nine obesity susceptibility SNPs with BMI and WC respectively were directionally consistent with results reported in the original GWA studies [13], [16], [17], [28]. The variants in *FTO* and near *MC4R* reached statistically significance for association with BMI. The locus in *FTO* was also statistically significant associated with WC. The GPS was associated with both BMI (B: 0.041 SD/allele [0.015, 0.067], p = 0.002) and WC (B: 0.033 SD/allele [0.008, 0.059], p = 0.011) (Table 3). Each additional BMI-increasing allele in the GPS increased the odds of overweight and obesity by respectively 1.08-fold [1.02–1.14], p = 0.006 and 1.08-fold [1.01–1.15], p = 0.045.

**Table 3:** Associations of the individual obesity-susceptibility SNPs and the GPS^1^ with adiposity-related traits^2^ in young adulthood (HUNT3)^3^.

				Z-scores BMI				Z-scores WC			
SNP	Chrom	Nearest gene	B	CI (95%)	P-value	Literature		B	CI (95%)	P-value	Literature
rs2815752	1	NEGR1	−.028	−.098, .042	.436		0.024^d^	−.007	−.077, .063	.845	0.022^d^
rs6548238	2	TMEM18	.018	−.075, .125	.699		0.070^d^	.017	−.076, .109	.726	0.050^d^
rs10938397	4	GNPDA2	−.001	−.071, .069	.976		0.045^d^	−.013	−.083, .057	.719	0.039^d^
rs987237	6	TFAP2B	.022	−.067, .111	.624		−	−.035	−.124, .054	.439	0.035^e^
rs4074134	11	BDNF	.054	−.034, .142	.226		0.055^d^	.072	−.016, .159	.109	0.049^d^
rs10838738	11	MTCH2	.048	−.022, .117	.182		0.021^d^	.062	−.007, .131	.080	0.011^d^
rs1121980	16	In FTO	.086	.017, .156	.015		0.086^d^	.071	.002, .140	.045	0.080^d^
rs17782313	18	MC4R	.103	.025, .181	.010		0.047^d^	.069	−.009, .147	.082	0.042^d^
rs11084753	19	KCTD15	.062	−.014, .138	.110		0.016^d^	.047	−.028, .123	.220	0.024^d^
GPS			.041	.015, .067	.002		0.039^d^	.033	.008, .059	.011	0.033^d^

1 The genetic predisposition score (GPS) is the sum of effect alleles from each of the nine individual SNPs.

2 Sex specific z-scores of BMI and waist circumference in young adulthood.

3 Number of participants: for individual SNPs = 1643 and for GPS = 1634 (those missing more than 3 SNPs excluded).

The linear regression models were adjusted for age regarding BMI and additionally also for height regarding WC, assuming an additive effect.

Pregnant participants were excluded.

Chrom: chromosome.

Comparable effect sizes in the literature:

d Li et al. [35];

e Lindgren et al. [19].

Given the strong prior evidence of association of the test SNPs with measures of obesity, we assessed the significance of associations at a nominal level without accounting multiple testing. If we had adjusted for multiple testing using a Bonferroni correction, associations reaching p<0.0055 would have been considered significant at the 5% α-level. As such, associations between genetic variants near *MC4R* and *KCTD15* and BMI and between near *MC4R* variant and WC in adolescence would be significant after accounting for multiple testing. The association of the GPS with adolescent BMI and WC and adult BMI would also reach significance.

### Longitudinal Analyses

The GPS showed no significant association with change in body fat estimates measured as the difference in z-score of BMI and WC between adolescence and young adulthood. Of the individual SNPs, only the variant in *MTCH2* showed an association with change in both BMI (B: 0.064 SD/allele [0.010, 0.118], p = 0.020) and WC (B: 0.085 SD/allele [0.019, 0.151], p = 0.011) (Table 4).

**Table 4:** Associations of the individual obesity susceptibility SNPs and the GPS^1^ with change in adiposity-related traits^2^ from adolescence into adulthood^3^.

			Delta BMI^2^			Delta WC^2^		
SNP	Chrom.	Nearest gene	Diff.DeltaZ	CI (95%)	P-value	Diff.DeltaZ	CI (95%)	P-value
rs2815752	1	NEGR1	−.009	−.064, .045	.734	.001	−.066, .067	.986
rs6548238	2	TMEM18	−.047	−.119, .025	.201	−.051	−.139, .036	.250
rs10938397	4	GNPDA2	−.011	−.066, .043	.685	−.041	−.107, .025	.224
rs987237	6	TFAP2B	−.018	−.087, .051	.604	−.028	−.112, .057	.521
rs4074134	11	BDNF	.025	−.043, .093	.406	.038	−.044, .121	.363
rs10838738	11	MTCH2	.064	.010, .118	.020	.085	.019, .151	.011
rs1121980	16	In FTO	.026	−.028, .080	.343	−.000	−.066, .066	.996
rs17782313	18	MC4R	−.048	−.108, .013	.125	−.064	−.138, .010	.090
rs11084753	19	KCTD15	−.038	−.097, .020	.201	−.005	−.077, .067	.891
GPS			−.003	−.023, .017	.762	−.004	−.029, .020	.726

1 The genetic predisposition score (GPS) is the sum of effect alleles from each of the nine individual SNPs.

2 Delta BMI and delta WC are differences between sex-specific z-scores in young adulthood and age-and-sex-specific z-scores in adolescence of BMI and WC respectively.

3 Number of participants: for individual SNPs = 1643 and for GPS = 1634 (those missing more than 3 SNPs excluded).

The linear regression models were adjusted for pubertal development and age-difference between adolescence and adulthood regarding change BMI and additionally also for height regarding change WC, assuming an additive effect. Pregnant participants were excluded.

Chrom: chromosome.

### Gene-physical Activity Interaction Effects

The effect of the GPS was somewhat larger in magnitude in the group adults who exercised less in their leisure time, but not so in adolescents. No significant interactions were found between physical activity with any of the individual SNPs or the GPS neither in the cross-sectional nor the longitudinal models (Tables S3, S4, and S5).

## Discussion

Our study, including 1 643 individuals with longitudinal data, showed that a GPS, based on nine established obesity-susceptibility loci, was significantly associated with BMI and WC in adolescence as well as in young adulthood. No significant associations between the GPS and change in BMI or WC between baseline and follow-up could be verified. Four of nine loci reached nominal significant associations when tested individually, whereas most of the associations of the five remaining loci showed directionally consistent associations. None of the interactions between physical activity and the obesity-susceptibility GPS could be statistically significant derived from our data, neither on the level nor the change in body fat estimates from adolescence into young adulthood.

Comparing our findings to those of others is difficult due to differences in age span, and selection of obesity-susceptibility variants. In spite of these differences, our data are in concordance with previous studies [5], [17], [20], [21], [30], also concluding that obesity-susceptibility loci found to be associated in adulthood, already affect anthropometric traits in adolescence. The GPS in our study showed an effect size in the same order of magnitude in relation to BMI in adolescence (B: 0.046) as den Hoed et al. [21] reported from the EYHS study (B: 0.044). In agreement with Zhao et al. [20], we found the locus near *MC4R* to be associated with BMI in adolescents.

We did not find an association between the GPS and change in body fat estimates from adolescence to adulthood. This might be due to the fact that our study had only one follow-up or did not capture enough time in the life course of the adolescents. Another reason might be that in concordance with Hardy et al. our data suggests that the genetic effects are slightly larger in adolescence and seem to decrease afterwards [5].

Li el al. [31], on the contrary, found an association between a GPS and change in BMI, but only in physically inactive participants. Haworth [32] discussed that the genetic influence on BMI becomes progressively stronger over childhood, because of stronger expression of genes (FTO) or due to the fact that children increasingly select environments which fit to their genetic propensities. Other studies [11], [12] have suggested that genetic variants, affecting BMI level, may be distinct from those affecting BMI change. This could maybe be supported by the finding that the single variant in *MTCH2* showed an association with change in both BMI and WC, not found at the cross sectional level. However, this finding was no longer statistically significant when controlling for multiple testing. Studies have reported different results for the attenuating effect of physical activity on obesity susceptibility markers [21], [31], [33], [34]. Examining a 12 BMI-loci GPS in a large study population, a previous study found an attenuating effect of physical activity on obesity susceptibility genes in adults [31]. A recent meta-analysis [34] reported that physical activity attenuated the influence of *FTO* variants on obesity risk in adults, but not in adolescents and children. Our data did not indicate any attenuating effect of physical activity on obesity susceptibility in adolescence or in young adulthood. This is consistent with the recent meta-analysis in regard to adolescents [34]. However, it might be noteworthy that our data all along the line indicated a larger effect size in the group of adolescents who exercised less in their leisure time. However, the differences did not reach statistical significance.

Due to the repeated cross sectional design of a total population used in HUNT and a low participation-rate for the age-group 23–29 year old in HUNT3, relatively few of the total number of participants in Young-HUNT 1 were followed-up. Comparisons of participants with non-participants at baseline (Young-HUNT 1) displayed no essential differences in the general characteristics (BMI, WC, gender), suggesting no major selection bias of the follow-up sample.

The main limitation to our study is the rather low number of participants and thus not finding statistical significance might be due to lack of power. Nevertheless, the associations for the four loci that that reach nominal significance are directionally consistent with those reported for theses loci in the original papers [17], [20], [21], [35] (i.e. the same allele increases obesity-susceptibility). Furthermore, most of associations for the loci that did not reach significance are also directionally consistent with those reported in the original paper. Therefore, we believe that our observations are not a mere reflection of chance. Larger sample sizes will be needed to firmly replicate (or refute) our observations.

This may be especially prominent when testing interaction effects. Besides, others have pointed out that replication studies may, besides providing insurance from the errors and biases that may be unavoidably afflict any study, also amplify confidence that any associations uncovered reflect processes that are biological interesting, rather than methodological inadequacies [36], [37].

In conclusion, we replicated partly the association between previously published obesity-susceptibility loci and adiposity-related traits in both adolescence and young adulthood. Loci known to affect obesity-susceptibility in adulthood already seem to do so in adolescence. Understanding the different mechanisms behind weight increase from childhood and adolescence to adulthood is important both in a clinical and prevention perspective. Our study might possibly show some indications of effects, which could not all be verified with statistically significance. More large population studies with focus on associations between genes and weight gain over time and possible attenuating effects as physical activity are needed.

## Supporting Information

Table S1 (DOCX)

Table S2 (DOCX)

Table S3 (DOCX)

Table S4 (DOCX)

Table S5 (DOCX)
