# Supplementary material for: Obesity-Susceptibility Loci and Their Influence on Adiposity-Related Traits in Transition from Adolescence to Adulthood - The HUNT Study
Source: PLoS One. 2012 Oct 19;7(10):e46912. doi: 10.1371/journal.pone.0046912 (PMC3477114; doi:10.1371/journal.pone.0046912)
Supplement: Table S1 — (DOCX) [file pone.0046912.s001.docx]

Table S1. Characteristics of participants in adolescence and young adulthood stratified by sex.

Adolescents (n=1634) Adults (n= 1634)

|  |  |  | Girls n=889 |  | Boys n=745 |  | Women |  | Men |  |
| --- | --- | --- | --- | --- | --- | --- | --- | --- | --- | --- |
| Age |  |  | 16.0 (1.8) |  | 15.9 (1.8) |  | 27.2 (1.9) |  | 27.4 (1.9) |  |
| BMI |  |  | 21.4 (3.2) |  | 21.1 (3.2) |  | 25.1 (4.8) |  | 26.1 (4.4) |  |
| Weight kg |  |  | 58.5 (9.9) |  | 64.0 (13.3) |  | 70.1 (13.8) |  | 84.8 (14.8) |  |
| Height cm |  |  | 165.2 (6.5) |  | 173.6 (9.5) |  | 167.0 (6.0) |  | 180.2 (6.5) |  |
| Waist circ. cm | |  | 70.3 (7.5) |  | 75.4 (8.4) |  | 85.4 (12.9) |  | 91.5 (11.5) |  |
| PDS | |  | 3.4 (0.6) |  | 3.0 (0.7) |  | / |  | / |  |
| Physical activity | | < 2 days/w | 33.3% |  | 30.4% |  | 40.2% |  | 42.5% |  |
|  | | ≥ 2 days/w | 66.7% |  | 69.6% |  | 59.8% |  | 57.5% |  |
|  | |  |  |  |  |  |  |  |  |  |

Data are means (SD). Participants who reported pregnancy at follow-up were excluded. PDS: pubertal development scale

≥2 days/w: physically activity equal or more than 2 days per week until they got out of breath or sweat.
