# Supplementary material for: Obesity-Susceptibility Loci and Their Influence on Adiposity-Related Traits in Transition from Adolescence to Adulthood - The HUNT Study
Source: PLoS One. 2012 Oct 19;7(10):e46912. doi: 10.1371/journal.pone.0046912 (PMC3477114; doi:10.1371/journal.pone.0046912)
Supplement: Table S2 — (DOCX) [file pone.0046912.s002.docx]

Table S2. Demographic characteristics at baseline (Young-HUNT 1) for the follow-up participants (n= 1634)

compared to the total Young-HUNT1 population (n=8408). Distributions in mean and (standard deviation), if not indicated otherwise.

|  |  |  | Girls |  |  |  | Boys |  |  |
| --- | --- | --- | --- | --- | --- | --- | --- | --- | --- |
|  |  | YH 1 |  | YH1H3 |  | YH1 |  | YH1H3 |  |
| n |  | 4179(49.7%) | | 889(54.4%) | | 4229(51.3%) | | 745 (45.6%) | |
| Age |  | 16.0(1.8) |  | 16.0(1.8) |  | 16.0(1.8) |  | 15.9(1.8) |  |
| BMI |  | 21.4(3.3) |  | 21.4(3.2) |  | 21.2(3.3) |  | 21.1(3.2) |  |
| WC |  | 70.6(8.0) |  | 70.3(7.5) |  | 75.8(8.8) |  | 75.4(8.4) |  |
| Phys. act |  | 3.5(1.6) |  | 3.5(1.6) |  | 3.3(1.8) |  | 3.4(1.7) |  |
|  |  |  |  |  |  |  |  |  |  |

YH1: Total Young-HUNT 1 population,

YH1H3: Participants in the follow-up study (participated in both Young-HUNT1 and HUNT 3).

BMI: body mass index in BMI units and WC (waist circumference) in centimetres.

Phys. act: outside of school, performing sports or exercise to the point of heavily breathing and/or sweating”. We assigned values 1 through 8 to the response options: Everyday, 4-6 days a week, 2-3 days a week; 1 day a week; Not every week, but at least once every two weeks; Not every 14^th^ day, but at least once a month; Less than once a month; Never.
