# Supplementary material for: Obesity-Susceptibility Loci and Their Influence on Adiposity-Related Traits in Transition from Adolescence to Adulthood - The HUNT Study
Source: PLoS One. 2012 Oct 19;7(10):e46912. doi: 10.1371/journal.pone.0046912 (PMC3477114; doi:10.1371/journal.pone.0046912)
Supplement: Table S3 — (DOCX) [file pone.0046912.s003.docx]

Table S3. Associations of the GPS^1^ with adiposity-related traits^2^ in adolescence of Young-HUNT1^3^ in different strata of physical activity at adolescence

|  | |  |  | Z-scores BMI | | |  |  | Z-scores WC | | | | | | | | | | |  |  |  |  |  |
| --- | --- | --- | --- | --- | --- | --- | --- | --- | --- | --- | --- | --- | --- | --- | --- | --- | --- | --- | --- | --- | --- | --- | --- | --- |
|  | | |  | B | CI (95%) | P-value |  |  | B | CI (95%) | | | | | | P-value | | | |  |  |  |  |  |
|  | |  |  |  |  |  |  |  |  |  | | | | | |  | | | |  |  |  |  |  |
| Physical activity | | < 2 days/w |  | 0.025 | -0.026 to 0.077 | 0.330 |  |  | 0.013 | -0.036 to 0.062 | | | | | | | | 0.604 | |  |  |  |  |  |
|  | | ≥ 2 days/w |  | 0.055 | 0.024 to 0.086 | 0.000 |  |  | 0.050 | 0.019 to 0.082 | | | | | | | | 0.002 | |  |  |  |  |  |
|  | |  |  |  | P interaction | 0.304 |  |  |  | P interaction | | | | | | | | 0.204 | |  |  |  |  |  |
|  | |  |  |  |  |  |  |  |  |  | | | | | | | |  | |  |  |  |  |  |
| ^1^ The genetic predisposition score (GPS) is the sum of effect alleles from each of the nine individual SNPs. | | | | | | | | | | | | | | |  | |  | |  | | |  | |  |
| ^2^ Age and sex specific z-scores of BMI and waist circumference in adolescence. | | | | | | | | | | |  |  |  |  |  | |  | |  | | |  | |  |
|  | ^3^ Number of participants: for GPS=1634 (those missing more than 3 SNPs excluded). | | | | | | | | | | | | | | | | | | |  | | |  | |
|  | The linear regression models were adjusted for pubertal maturity regarding BMI and additionally also for height regarding WC, assuming an additive effect. Pregnant participants were excluded.  ≥2 days/w: physically activity in adolescence was doing exercise equal or more than 2 days per week until they got out of breath or sweat. | | | | | | | | | | | | | | | | | | | | | | | |
