# Supplementary material for: Obesity-Susceptibility Loci and Their Influence on Adiposity-Related Traits in Transition from Adolescence to Adulthood - The HUNT Study
Source: PLoS One. 2012 Oct 19;7(10):e46912. doi: 10.1371/journal.pone.0046912 (PMC3477114; doi:10.1371/journal.pone.0046912)
Supplement: Table S4 — (DOCX) [file pone.0046912.s004.docx]

Table S4. Associations of the GPS^1^ with adiposity-related traits^2^ in young adulthood (HUNT3)^3^ in different strata of physical activity at adulthood.

|  | |  | | |  | |  |  | | Z-scores BMI | | | | | | |  | Z-scores WC | | |  |  |
| --- | --- | --- | --- | --- | --- | --- | --- | --- | --- | --- | --- | --- | --- | --- | --- | --- | --- | --- | --- | --- | --- | --- |
|  | | | | |  | |  | B | | | CI (95%) | P-value | |  | | |  | B | CI (95%) | P-value |  |  |
|  | |  | | |  | |  |  | | |  |  |  | |  |  | | |  |  |  |  |
| Physical activity | | < 2 days/w | | |  | |  | 0.055 | | | 0.011 to 0.098 | 0.014 |  | |  | 0.040 | | | -0.003 to 0.083 | 0.071 |  |  |
|  | | ≥ 2 days/w | | |  | |  | 0.029 | | | -0.006 to 0.064 | 0.104 |  | |  | 0.027 | | | -0.008 to 0.062 | 0.130 |  |  |
|  | |  | | |  | |  |  | | | P interaction | 0.685 |  | |  |  | | | P interaction | 0.589 |  |  |
|  |  | |  |  | |  | | |  |  |  |  |  |  |  |  |  |  |  |  |  |  |

^1^ The genetic predisposition score (GPS) is the sum of effect alleles from each of the nine individual SNPs

^2^ Sex specific z-scores of BMI and waist circumference in young adulthood.

^3^ Number of participants: for GPS=1634 (those missing more than 3 SNPs excluded)

The linear regression models were adjusted for age regarding BMI and additionally also for height regarding WC, assuming an additive effect

Pregnant participants were excluded.

≥2 days/w: physically activity in adulthood was doing exercise equal or more than 2 days per week until they got out of breath or sweat.
