# Supplementary material for: Obesity-Susceptibility Loci and Their Influence on Adiposity-Related Traits in Transition from Adolescence to Adulthood - The HUNT Study
Source: PLoS One. 2012 Oct 19;7(10):e46912. doi: 10.1371/journal.pone.0046912 (PMC3477114; doi:10.1371/journal.pone.0046912)
Supplement: Table S5 — (DOCX) [file pone.0046912.s005.docx]

Table S5. Associations of the GPS^1^ with change in adiposity-related traits^2^ from adolescence into adulthood^3^ in different strata of physical activity at adolescence.

________________________________________________________________________________________________________________________

|  |  |  |  | Delta BMI^2^ | | |  | | Delta WC^2^ | | |  |
| --- | --- | --- | --- | --- | --- | --- | --- | --- | --- | --- | --- | --- |
|  |  |  |  |  |  |  |  |  | |  |  |  |
| GPS |  |  |  | Diff.DeltaZ | CI (95%) | P-value |  | Diff.DeltaZ | | CI (95%) | P-value |  |
|  |  |  |  |  |  |  |  |  | |  |  |  |
| Physical activity | < 2 days/w |  |  | 0.008 | -0.032 to 0.048 | 0.686 |  | 0.008 | | -0.038 to 0.053 | 0.741 |  |
|  | ≥ 2 days/w |  |  | -0.008 | -0.031 to 0.015 | 0.515 |  | -0.010 | | -0.039 to 0.020 | 0.522 |  |
|  |  |  |  |  | P interaction | 0.475 |  |  | | P interaction | 0.448 |  |

^1^ The genetic predisposition score (GPS) is the sum of effect alleles from each of the nine individual SNPs.

^2^ Delta BMI and delta WC are differences between sex-specific z-scores in young adulthood and age-and-sex-specific z-scores in adolescence of BMI and WC respectively.

^3^ Number of participants: for GPS=1634 (those missing more than 3 SNPs excluded).

The linear regression models were adjusted for pubertal development and age-difference between adolescence and adulthood regarding change BMI and additionally also for height regarding change WC, assuming an additive effect. Pregnant participants were excluded.

≥2 days/w: physically activity in adolescence was doing exercise equal or more than 2 days per week until they got out of breath or sweat
